# Supplementary material for: Content-rich biological network constructed by mining PubMed abstracts
Source: BMC Bioinformatics. 2004 Oct 8;5:147. doi: 10.1186/1471-2105-5-147 (PMC528731; doi:10.1186/1471-2105-5-147)
Supplement: Additional File 2 — The original results of the above study (non-essential files are deleted to keep the file size under the limit set by BMC bioinformatics). [file 1471-2105-5-147-S2.bz2 › chilibotAdditionalFile2/dip30/693ID11994151E18018/html/TOLA_TOLB.html]

 


 **TOLA** and **TOLB** 
  
Found 36 abstracts in PubMed, retrieved 30.  
 

 What does Google say? 
 PDF only 
| .edu only 

---

**Interactive relationship** (e.g. stimulation, inhibition, etc)

**Neutral relationship**- Mutational analysis of the  **TolA**  C terminal domain of Escherichia coli and genetic evidence for an interaction between  **TolA**  and  **TolB** .  Ref: 12142433 J Bacteriol, 2002
- The  **TolA**   **TolB**  interaction was confirmed by cross linking experiments on purified proteins.  Ref: 11994151 Mol Microbiol, 2002
- These results demonstrate that the  **TolA**   **TolB**  interaction allows the formation of a trans envelope complex that brings the inner and outer membranes in close proximity.  Ref: 11994151 Mol Microbiol, 2002
- Moreover, we show that the interaction between  **TolA**  and  **TolB**  is required for the uptake of colicin A and for the membrane integrity.  Ref: 11994151 Mol Microbiol, 2002
- Distinct regions of the colicin A translocation domain are involved in the interaction with  **TolA**  and  **TolB**  proteins upon import into Escherichia coli.  Ref: 9466263 Mol Microbiol, 1998
- **TolB**  dimerizes and the periplasmic domain of  **TolA**  interacts with YbgF and  **TolB** .  Ref: 11994151 Mol Microbiol, 2002
- This region and the other regions responsible for the interaction with  **TolA**  and  **TolB**  have been mapped precisely within the colicin A N terminal domain and appear to be arranged linearly in the colicin sequence.  Ref: 11703658 Mol Microbiol, 2001
- The Tol Pal system function requires an interaction between the C terminal domain of  **TolA**  and the N terminal domain of  **TolB** .  Ref: 11994151 Mol Microbiol, 2002
- Our results indicate that the central domain of  **TolA**  TolAII is sufficient to interact with YbgF, that the C terminal domain of  **TolA**  TolAIII is sufficient to interact with  **TolB** ,.  Ref: 11994151 Mol Microbiol, 2002
- These results suggest that the interactions of  **TolA**  and  **TolB**  with porins might take place in vivo and might be concomitant events participating in porin assembly.  Ref: 9393690 J Bacteriol, 1997
- Transmembrane domains of TolQ, TolR and  **TolA**  interact in the cytoplasmic membrane, while  **TolB**  and Pal form a complex near the outer membrane.  Ref: 9701827 Mol Microbiol, 1998
- Transmembrane domains of TolQ, TolR, and  **TolA**  interact in the cytoplasmic membrane, while  **TolB**  and Pal form a complex near the outer membrane.  Ref: 9851983 J Bacteriol, 1998
- The isolation of suppressor mutants of  **tolA**  mutations in the  **tolB**  gene confirmed an interaction between TolAIII and the N terminal domain of  **TolB** .  Ref: 12142433 J Bacteriol, 2002

**Non-interactive relationship** (e.g. studied together, co-existance, homology, etc.)

- The nucleotide sequence of a 2.8 kilobase fragment containing the  **tolA**  and  **tolB**  genes was determined.  Ref: 2687247 J Bacteriol, 1989
- Bacteriocin sensitivity tests performed on a collection of E. coli envelope mutants, and isolation and characterization of E. coli bacteriocin 28b insensitive mutants, showed that the core lipopolysaccharide, outer membrane proteins OmpA and OmpF, and TolQ,  **TolA** , and  **TolB**  proteins are involved in bacteriocin 28b lethal activity.  Ref: 8595593 Can J Microbiol, 1996
- The colicin A N terminal domain was also shown to form a trimeric complex with  **TolA**  and  **TolB** .  Ref: 9466263 Mol Microbiol, 1998
- The N terminal domains of colicins A and E1 have been shown to interact with  **TolA** , and the N terminal domain of colicin E3 has been shown to interact with  **TolB** .  Ref: 9466263 Mol Microbiol, 1998
- **TolA** , TolQ, and TolR are localized in the inner membrane while  **TolB**  is periplasmic, although a small amount of membrane associated  **TolB**  is always found.  Ref: 7744737 J Biol Chem, 1995
- Strains of Escherichia coli K12 carrying a  **tolA** ,  **tolB** , lky or exc mutation located at min 16.5 on the genetic map released periplasmic proteins into the extracellular medium.  Ref: 2573819 Mol Gen Genet, 1989
- The  **TolA** ,  **TolB** , TolQ, and TolR proteins are involved in maintaining the integrity of the Escherichia coli outer membrane and in the import of group A colicins and filamentous phage DNA.  Ref: 7744737 J Biol Chem, 1995
- This cluster was found to consist of seven genes in the order orf1 tolQ tolR  **tolA**   **tolB**  oprL orf2.  Ref: 10735848 J Bacteriol, 2000
- We consider a variety of different colicins, including A, B, D, E1, E3, Ia, M and N, that penetrate through the porins OmpF, FepA, BtuB, Cir and FhuA, to subsequently interact with a few targets in the periplasm, including  **TolA** ,  **TolB** , TolC and TonB.  Ref: 12423783 Biochimie< MedlineTA>Biochimie, 2000
- Mutations in tolQ, tolR,  **tolA** ,  **tolB** , or pal genes result in sensitivity to bile salts and the leakage of periplasmic proteins.  Ref: 10633120 J Bacteriol, 2000
- **TolB**  and the central domain of  **TolA**  interact in vitro with the outer membrane porins.  Ref: 9701827 Mol Microbiol, 1998
- In the present study we cloned and mutagenized the orf1, tolQ, tolR,  **tolA** , and  **tolB**  genes from P. putida KT2440, which were located upstream of the oprL gene.  Ref: 10940016 J Bacteriol, 2000
- It is organized into two complexes, one near the outer membrane between  **TolB**  and PAL and one in the cytoplasmic membrane between  **TolA** , TolQ, and TolR.  Ref: 10419942 J Bacteriol, 1999
- The Tol Pal system of Escherichia coli is composed of the YbgC, TolQ,  **TolA** , TolR,  **TolB** , Pal and YbgF proteins.  Ref: 11994151 Mol Microbiol, 2002
- Group A colicins need proteins of the Escherichia coli envelope Tol complex  **TolA** ,  **TolB** , TolQ and TolR to reach their cellular target.  Ref: 9466263 Mol Microbiol, 1998
- The interactions of colicin A N terminal domain deletion mutants with  **TolA**  and  **TolB**  were investigated.  Ref: 9466263 Mol Microbiol, 1998
- The deduced amino acid aa sequence of the H. influenzae TolQ, TolR,  **TolA**  and  **TolB**  show 6 1 and 62% identity with Escherichia coli TolQRAB proteins, respectively.  Ref: 8921895 Gene, 1996
- In this study, we demonstrate that the genes tolQ, tolR, and  **tolA** , but not  **tolB** , tolC, and ompF, are required for the internalization of cloacin DF13 and they are not involved in the transport of ferric aerobactin.  Ref: 8419302 J Bacteriol, 1993
- Uptake of the mutated colicin U DTMVV depended on TonB and ExbB and still remained  **TolA**  and TolQ dependent, but no longer required  **TolB** .  Ref: 9682486 FEMS Microbiol Lett, 1998
- **TolA** , TolQ, and TolR are inner membrane proteins,  **TolB**  is a periplasmic protein, and Pal, the peptidoglycan associated lipoprotein, is anchored to the outer membrane.  Ref: 11790745 J Bacteriol, 2002
- Subcloning experiments and complementation studies showed that lky and exc mutations were located either in the previously described  **tolA**  and  **tolB**  genes or in the newly characterized excC and excD genes.  Ref: 2573819 Mol Gen Genet, 1989
- Analysis of polar mutations, reverse transcriptase PCR assays, and transcriptional fusion constructs with a promoterless lacZ gene revealed that the genes are arranged in two operons orf1 tolQ tolR  **tolA**   **tolB**  and oprL orf2.  Ref: 12486055 J Bacteriol, 2003
- The Tol Pal system of the Escherichia coli envelope is formed from the inner membrane TolQ, TolR and  **TolA**  proteins, the periplasmic  **TolB**  protein and the outer membrane Pal lipoprotein.  Ref: 11115123 Mol Microbiol, 2000
- Our results demonstrate that different regions of the colicin A N terminal domain interact with  **TolA**  and  **TolB** .  Ref: 9466263 Mol Microbiol, 1998
- Colicin A interacts with  **TolA**  and  **TolB**  via distinct regions of its N terminal domain.  Ref: 11703658 Mol Microbiol, 2001
- In the presence of the purified central domain of  **TolA**  TolAIIHis, the  **TolB**  porin complexes disappeared to form TolAIIHis porin complexes.  Ref: 9393690 J Bacteriol, 1997
- Polar and nonpolar mutations of the P. putida tolQ, tolR,  **tolA** , and  **tolB**  genes were generated in vitro by using the omega Km r interposon, which carries two transcriptional stop signals, or a promoterless xylE cassette, lacking any transcriptional stop signal, respectively.  Ref: 10940016 J Bacteriol, 2000
- The TolQ, TolR,  **TolA** ,  **TolB** , and Pal proteins appear to function in maintaining the integrity of the outer membrane, as well as facilitating the uptake of the group A colicins and the DNA of the infecting filamentous bacteriophages.  Ref: 8763928 J Bacteriol, 1996
- The TolQ TolR  **TolA**  proteins form a complex in the cytoplasmic membrane, while  **TolB**  and Pal interact near the outer membrane.  Ref: 10474183 FEMS Microbiol Lett, 1999
- In Pseudomonas putida, the seven genes, orf1, tolQ, tolR,  **tolA** ,  **tolB** , oprL, and orf2, which encode the proteins of this complex, are clustered in a 5.8 kb region of chromosomal DNA.  Ref: 12486055 J Bacteriol, 2003
- **TolA**  and TolR are anchored to the inner membrane by a single transmembrane domain, TolQ is an integral membrane protein with three transmembrane segments, and  **TolB**  has recently been found to be periplasmic although it is partially membrane associated.  Ref: 7744736 J Biol Chem, 1995
- Nucleotide sequences of the  **tolA**  and  **tolB**  genes and localization of their products, components of a multistep translocation system in Escherichia coli.  Ref: 2687247 J Bacteriol, 1989
- Most of the amino acid residues of  **TolA** ,  **TolB** , TolR and Pal are localized in the periplasm.  Ref: 10474183 FEMS Microbiol Lett, 1999
- Analysis of double mutants strains carrying mutation staA 2 and a  **tolA** ,  **tolB** , excC or excD periplasmic leaky mutation showed that staA suppression was allele specific which suggested that proteins  **TolA**  and StaA might directly interact.  Ref: 2040438 FEMS Microbiol Lett, 1991
- Isolation and characterization of the Haemophilus influenzae tolQ, tolR,  **tolA**  and  **tolB**  genes.  Ref: 8921895 Gene, 1996
- Sequence data showed that these genes are clustered in a 6 kb segment of DNA with the gene order orf1 tolQ tolR  **tolA**   **tolB**  pal orf2 a newly identified open reading frame encoding a 29 kD9 protein.  Ref: 8763928 J Bacteriol, 1996
- Furthermore, bacteriocin 28b translocation requires proteins  **TolA** ,  **TolB** , and TolQ.  Ref: 8595593 Can J Microbiol, 1996
- The predicted amino acid sequences of V. cholerae TolQ, TolR,  **TolA** , and  **TolB**  showed significant similarity to the corresponding E. coli sequences.  Ref: 10692381 J Bacteriol, 2000
